# National Medical Union

**Published:** 1918-06-08

**Authors:** 


### National Medical Union.

THE annual general meeting of the National Medical Union, presided over by Professor William Russell, President of the Royal College of Physicians, Edinburgh, was held on Saturday, June 1, at 346 Strand, London. The meeting was well attended, and the following resolutions were unanimously agreed to :—

1. "That this annual meeting of the National Medical Union advises its members not to sign any application for exemption under the Second Military Service Act, as to do so may render them liable to be transferred to other localities for civilian practice or to be compelled to undertake panel practice in their own localities."

2. "That the National Medical Union restates its contention that the establishment of a Ministry of Health ought to be postponed until after the war, and protests against a propaganda directed towards the preparation of an atmosphere in the public mind favourable to an establishment of general State Medical Service, such a general State Medical Service being in the opinion of the Union an unwarrantable interference on the part of the Government with the relation which exists between the public and members of the medical profession."

The meeting also unanimously confirmed and adopted the resolution of its Council approving of the principle of compulsory medical service of purely military nature, but strongly condemning any attempt to extend compulsion in respect of civilian practice.

